# Deciphering *Aphanomyces euteiches*-pea-biocontrol bacterium interactions through untargeted metabolomics

**DOI:** 10.1038/s41598-024-52949-w

**Published:** 2024-04-17

**Authors:** Zakir Hossain, Shuang Zhao, Xian Luo, Kui Liu, Liang Li, Michelle Hubbard

**Affiliations:** 1 Swift Current Research and Development Centre, Agriculture and Agri-Food Canada, 1 Airport Road, Swift Current, Saskatchewan, S9H 3X2 Canada; 2 Department of Chemistry, University of Alberta, Edmonton, AB T6G 2G2 Canada

**Keywords:** Chemical biology, Microbiology, Plant sciences

## Abstract

*Aphanomyces euteiches* causes root rot in pea, leading to significant yield losses. However, the metabolites involved in this pathosystem have not been thoroughly studied. This study aimed to fill this gap and explore mechanisms of bacterial suppression of *A. euteiches* via untargeted metabolomics using pea grown in a controlled environment. Chemical isotope labeling (CIL), followed by liquid chromatography-mass spectrometry (LC–MS), was used for metabolite separation and detection. Univariate and multivariate analyses showed clear separation of metabolites from pathogen-treated pea roots and roots from other treatments. A three-tier approach positively or putatively identified 5249 peak pairs or metabolites. Of these, 403 were positively identified in tier 1; 940 were putatively identified with high confidence in tier 2. There were substantial changes in amino acid pool, and fatty acid and phenylpropanoid pathway products. More metabolites, including salicylic and jasmonic acids, were upregulated than downregulated in *A. euteiches*-infected roots. 1-aminocyclopropane-1-carboxylic acid and 12-oxophytodienoic acid were upregulated in *A. euteiches* + bacterium-treated roots compared to *A. euteiches*-infected roots. A great number of metabolites were up- or down-regulated in response to *A. euteiches* infection compared with the control and *A. euteiches* + bacterium-treated plants. The results of this study could facilitate improved disease management.

## Introduction

Plants are frequently exposed to environmental factors, including biotic adversaries such as bacteria, fungi, oomycetes, viruses, herbivores, and nematodes. Metabolites, associated with plant trait development^1^, are critical for plant responses to these stresses^2,3^. Plants produce a large array of secondary or specialized metabolites (SMs)^2^, including phenylpropanoids, flavones, and phytoalexins, that play important roles in protecting plants from pathogen infection^4,5^. Exposure to biotic stresses changes plant defense-related metabolites level or induce the production of specific SMs that modulate plant responses to invading pathogens and enhance immunity^5,6^.

Root rot of pea caused by *Aphanomyces euteiches* Drechs. can reduce pea yields by up to 80%^7^. Effective tools to manage this disease are lacking. Thus, alternative approaches, such as biological control, are of interest. A better understanding of the metabolites at play in *A. euteiches* infection of pea, with and without a potential biocontrol agent, would be both scientifically novel and of practical value. For example, it could facilitate resistant variety development and improvement of potential biocontrol agents.

Induced systemic resistance (ISR) is a major component of the plant immune system^8^. ISR is regulated by a network of interconnected signaling pathways, including plant hormones such as jasmonic acid (JA) and ethylene (ET). Plants can develop ISR in response to infection by a pathogen and/or colonization of the roots by beneficial microbes^8^. Trichoderma, a well-studied beneficial microbe, contributes to ISR induction in response to infection with different soil-borne pathogens by modulating plant defense-related metabolites such as polyphenols, flavonoids, and terpenes levels in crops^9,10^. In *Arabidopsis*, Trichoderma-induced ISR in response to *Botrytis cinerea* infection resulted in changes in the levels of phytohormones—such as JA, salicylic acid (SA)—and other metabolites^11,12^. However, little is known about the root metabolome in legumes colonized by beneficial microbes.

Metabolomics can identify the metabolic changes within an organism^13^, characterize the pathways that regulate those changes, and detect active metabolites related to phenotype^14^. It therefore allows qualitative and quantitative measurement of metabolites in plants challenged by pathogens. Although metabolomics has been successfully used to investigate plant–microbe interactions in recent years^15,16^, use of this technology is limited in annual legume crops. Despite being an important legume crop worldwide, none or little metabolomics studies has been conducted on pea-pathogen interaction; particularly no metabolomic information is available on pea plants infected with *A. euteiches*.

Many beneficial microorganisms, such as plant growth promoting rhizobacteria (PGPR), are antagonistic to pathogens^8^. Plants also use metabolites to influence the composition of the microbiome in the rhizosphere^3,17^. However, information on the biochemical and molecular mechanisms of tripartite interactions, plant-pathogen-beneficial microbe, are scarce. During the interaction of a pathogen, a beneficial microbe, and the host plant, each of their respective metabolomes are likely altered due to the influx of exogenous biomolecules from the other two participants^18,19^. Alteration of metabolites in various biochemical pathways may have significant impact on plant physiology.

Because of the complexity of interactions, the choice of analytical technique is critical in investigating plant–microbe interactions. Liquid chromatography separation coupled with high-resolution mass spectrometry (LC–MS) can detect metabolites with diverse chemical properties with high mass accuracy. Plant–microbe interactions might generate a plethora of unknown biologically active chemical compounds; an untargeted metabolomics approach would be suitable to identify and annotate a greater number of metabolites. Chemical isotope labeling (CIL) LC–MS is a relatively recently developed method used in relative quantification for untargeted metabolomics and absolute quantification for targeted metabolomics^20^.

In this study, using an untargeted metabolomics approach, we attempted to identify metabolic changes due to pea-*A. euteiches* and pea-*A. euteiches*-bacterium interactions. We used CIL LC–MS to conduct untargeted metabolomics, focusing on metabolites that are likely to be involved in plant defense responses, in pea roots with or without inoculation with *A. euteiches* and/or a biocontrol bacterial isolate PD-S66. This isolate was selected for its capacity to suppress *A. eutieches* growth in vitro and aphanomyces root rot in pea in pot trials. In addition to co-infection with *A. euteiches* and the bacterium, we inoculated pea with *A. euteiches* and bacterium separately. To facilitate determining whether metabolites originate from the plant, the pathogen, or the bacteria, we included pure *A. euteiches* and bacterial culture in the analysis. We hypothesized that the host–pathogen and host–pathogen–bacterium interactions would lead to different biochemical responses, resulting in the changes in the levels of defense-related metabolites or to the induction of distinct metabolite(s). We therefore designed this study to investigate the mechanisms of (1) the interaction between pea and *A. euteiches*, and (2) bacterial suppression of the pathogen by observing the changes in metabolites levels in pea roots. The results provided novel insight into the mechanisms of *A. euteiches* infection of pea root and of bacterial biocontrol.

## Materials and methods

### Pathogen and bacterial culture

**Figure 1:**
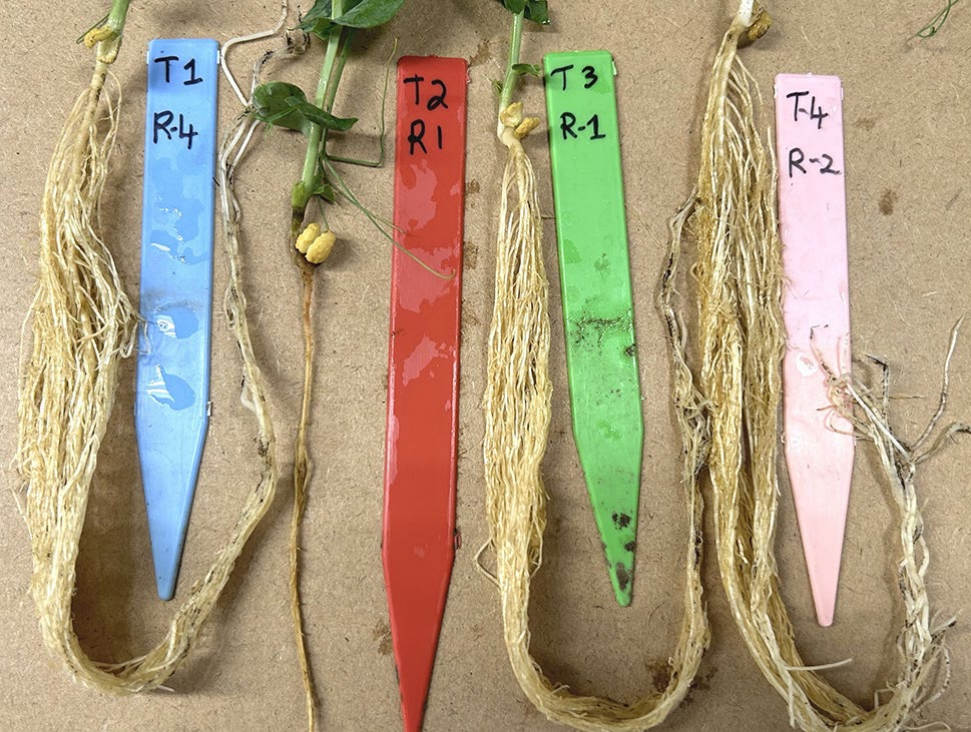
Effectiveness of PD-S66 in suppressing aphanomyces root rot in pea in the growth chamber (plants at 20-days post-inoculation).

For *A. euteiches* oospore production, three agar pieces (1 cm^2^ each) from an actively growing, 5-day old, culture of isolate Ae SK-2015 on corn meal agar, were transferred to a 250 mL Erlenmeyer flask containing 50 mL of sterile oatmeal broth (5 g oatmeal L^−1^). The flasks were incubated at 24 °C for 30 days in the dark to induce oospore production. The cultures were then pooled (typically from 10 flasks) in sterile conditions and homogenized for 4 × 1 min with a TissueRuptor II (QIAGEN) at medium speed. The oospore suspension was passed through 4-layers of sterile cheesecloth and counted under a compound light microscope using a haemocytometer. The pure *A. euteiches* culture was produced in five mL of potato dextrose broth inoculated with actively growing pathogen from two 0.5 × 0.5 cm agar pieces. The broth was incubated at 24 °C for 7 days in darkness. A pure culture of the select bacterium [PD-S66, *Pseudomonas* sp., selected for its capacity to suppress aphanomyces root rot on pea in pot trials (Fig. 1)] was produced in Luria Bertani broth, placed in an incubator for 20 h at 30 °C with shaking at 200 rpm.

### Plant materials and sample collection

Three separate experiments were conducted in a growth chamber [16/8 h light (200 µmol photons m^−2^ s^−1^)/darkness, 24 °C during the day and 21 °C at night temperature, and 75% relative humidity] using an aphanomyces root rot susceptible field pea variety, CDC Meadow. Seeds were sown in autoclaved field soil collected from Swift Current Research and Development Centre farm in 2 L pots with four replications. The *A. euteiches* oospore suspension was directly applied on the seeds (3 mL containing ~ 50,000 oospores mL^−1^ into each pot) followed by the addition of 2.5 mL bacterial suspension (~ 2.5 × 10^9^ CFU mL^−1^) on top of the pathogen (T4, PBBT), and then covered with soil. Plants without pathogen or bacterium served as the negative control (T1, control), while plants treated only with the pathogen (T2, PT) were the positive control. Plants treated only with the bacterium (T3, BBT) were also included to evaluate the impact of the bacterium on pea metabolites. The pots were watered daily to keep soil moist to favor disease development. Root samples were collected for metabolite extraction as soon as mild symptoms developed on the positive control. This occurred at 16-days post-inoculation. Plants were carefully dug up and roots were washed quickly, but gently, under running tap water. Roots from 4 to 6 seedlings were detached from the shoots with a sharp sterile blade, flash frozen in liquid nitrogen, and ground into fine powder in a mortar and pestle (baked at 200 °C for four hours). About 300 mg of powder was transferred into 2-mL Eppendorf tube (in duplicate). The powder was not allowed to thaw at any point of the procedure. Finally, tubes were stored at − 80 °C until analysis.

### Chemicals and reagents

All the chemicals and reagents, unless otherwise stated, were from Sigma-Aldrich Canada (Markham, ON, Canada). The CIL reagents were from Nova Medical Testing, Inc. (NovaMT, Edmonton, AB, Canada). LC–MS grade water, acetonitrile (ACN), and methanol (MeOH) were from Thermo Fisher Scientific (Edmonton, AB, Canada).

### Metabolite extraction

500 μL ice-cold 4:1 (v/v) MeOH: H_2_O solvent was added into each of the vials containing pea root powder. The vials were vortexed with 2.8 mm diameter ceramic beads for 15 s on a Bioprep-24 homogenizer. The samples were then centrifuged at 15,000*g* for 10 min at 4 °C. The supernatants were transferred into new vials and dried down with a SpeedVac.

200 μL 1:1 (v/v) MeOH: H_2_O solvent was added into each of the vials containing bacteria or pathogen. The vials were sonicated in an ice-water bath for 10 min before being centrifuged at 15,000*g* for 10 min at 4 °C. The supernatants were transferred into new vials and dried down. All the samples were re-dissolved in chromatography-grade H_2_O before analysis.

### Sample normalization and aliquoting

The total concentrations of samples were determined by NovaMT Sample Normalization kit. For the samples having total concentration greater than 2 mM, water was added to adjust their concentrations to 2 mM. For each sample, one aliquot of 25 μL was taken for amine and phenol labeling, one aliquot of 25 μL was taken for carboxyl labeling, and another 50 μL was taken from each sample to generate pooled samples for each type of samples (i.e., plant, bacteria, and pathogen).

### Chemical isotope labeling

Samples were labelled in vitro using specific reagent(s) to target amine, phenol, and carboxyl groups as these groups combined are likely to cover large proportions of metabolites associated with plant–microbe interactions. In this approach, individual samples were labeled with a light isotope reagent (such as ^12^C-dansyl chloride (DnsCl)), while a pooled sample, working as the reference and internal standard, was labeled with a heavy isotope reagent (such as ^13^C-DnsCl). In principle, all the metabolites with same functional group should react with the reagent and form the corresponding derivatized metabolites^20^. In the mass spectra, each metabolite is detected as a peak pair, containing a light peak (light-labeled derivative) and a heavy peak (heavy-labeled derivative). The intensity ratio of light and heavy peaks is used for relative quantification of metabolites.

The labeling experiments were performed by using the NovaMT labeling kits and following the NovaMT standard operating protocols for amine/phenol labeling and acid labeling. Briefly, 10 μL of buffer reagent (Reagent A, 250 mM sodium carbonate/sodium bicarbonate buffer) and 37.5 μL of freshly prepared ^12^C-DnsCl solution (18 mg/mL) were used for light labeling of individual and pooled samples. For pooled samples, 10 μL of buffer and 37.5 μL of freshly prepared ^13^C-DnsCl solution (18 mg/mL) were used. The samples were then vortexed, followed by spinning to get the mixture at the bottom of the tube. The mixtures were incubated at 40 °C for 45 min. After that, 7.5 μL of 250 mM sodium hydroxide solution (Reagent C) was added to quench the excessive labeling reagent. The mixtures were incubated at 40 °C for another 10 min. Finally, 30 μL of 425 mM formic acid in 1:1 ACN/H_2_O (Reagent D) was added to neutralize the remaining NaOH and make the solution acidic.

For carboxyl labeling, each aliquot of sample was dried down by nitrogen concentrator. The dried extracts were then re-dissolved with 25 μL of 3:1 (v/v) ACN: H_2_O. For labeling, 10 μL of catalyzing reagent (Reagent A) and 25 μL of ^12^C-* p*-Dimethylaminophenacyl Bromide (DmPABr) (for the individual samples and the pooled sample) or ^13^C-DmPABr (for the pooled sample) reagent (Reagent B) was added into samples. The samples were then vortexed, and subsequently spun down. The mixtures were incubated at 80 °C for 60 min and then spun down. After that, the excess labeling reagent was quenched by adding 40 μL of quenching reagent (Reagent C). The mixtures were incubated at 80 °C for another 30 min to complete the labeling procedure.

### Mixing

Equal volumes of a ^12^C-DnsCl-labeled individual sample was mixed with a ^13^C-DnsCl-labeled reference sample for each type of samples. For example, for bacteria samples, a ^12^C-DnsCl-labeled sample from each bacteria sample was mixed with a ^13^C-DnsCl-labeled bacterial samples from all bacterial samples pooled together. This mixture was used for LC–MS analysis. Prior to LC–MS analysis of the entire sample set, a quality control (QC) sample was prepared by mixing an equal volume of a ^12^C-DnsCl-labeled plant pooled samples (i.e., excluding the samples from the bacteria or *A. euteiches*) and a ^13^C-DnsCl-labeled plant pooled sample.

### LC–MS analysis

The ^12^C-/^13^C-labeled mixtures of each sample were analyzed using a 1290 ultra-high performance liquid chromatography instrument (UHPLC, Agilent) linked to a Bruker Impact II quadrupole time-of-flight (QTOF) mass spectrometer (Bruker, Billerica, MA) with electrospray ionization (ESI). The separation was performed using an Agilent Eclipse Plus reversed-phase C18 column (2.1 mm × 150 mm, 1.8 μm particle size). Mobile phase A was 0.1% (v/v) formic acid in water. Mobile phase B was 0.1% (v/v) formic acid in acetonitrile (ACN). The gradient for the separation was: t = 0 min, 25% B; t = 10 min, 99% B; t = 13 min, 99% B; t = 13.1 min, 25% B; t = 16 min, 25% B. All the samples were injected in random order. QC samples were injected every 10 sample runs to monitor instrument performance. The flow rate was 400 μL min^−1^ and the sample injection volume was 2 μL. The column temperature was 40 °C. All MS spectra were collected at a mass scan range of m/z 220–1000 at a spectral acquisition rate of 1 Hz in positive ion mode.

### Data processing and cleansing

A total of 66 LC–MS datasets (including retention times, m/z values, and peak intensities) from 2-channel (i.e., amine/phenol channel and acid channel) analyses (33 LC–MS dataset, including 3 QC in each channel) were first exported as .csv files with Bruker Data Analysis software (Bruker DataAnalysis 4.4). The exported data were uploaded to IsoMS Pro 1.2.10 (Nova Medical Testing Inc., Edmonton, AB, Canada)^21^ for format conversion, data quality check, and data processing. During data processing, redundant peaks such as dimers and adduct ions were filtered out. After aligning peak pairs from multiple samples using the alignment program, the Zerofill program^22^ was used to retrieve missing values that might have been lost during the previous data processing steps due to low signal intensity (i.e., below the detection limit) with a rationally determined ratio.

Seven groups were assigned to 33 LC–MS datasets in each channel. Each of control, PT, BBT, and PBBT group contained six data files. Each of bacterium (Bac), *A. euteiches* (Patho), and quality control (QC) group contained three data files. Peak pairs present in at least 80% of samples in any group were retained for further analysis. Two-channel LC–MS data from each sample were combined after processing. Peak ratio data were normalized by the ratio of total useful signals for each metabolite peak pair.

### Metabolite identification

Metabolite identification was carried out at three different levels of confidence (three tiers) using IsoMS Pro software and database. In tier 1, detected metabolites were positively identified by matching against a chemical isotope labeling library (CIL Library; amine/phenol and carboxyl channels) standards based on accurate mass and retention time. In tier 2, a metabolite (peak pair) was putatively identified with high confidence based on accurate mass and predicted retention time by comparing against a Linked Identity library (LI Library). The LI Library includes metabolic pathway-related metabolites with over 7000 entries extracted from the KEGG database. Thus, it provides high-confidence putative identification. Metabolites identification in tier 3 were based on accurate mass searches against compound entries in MyCompoundID (MCID) (http://www.mycompoundid.org/) library (zero-reaction library), and their predicted metabolic products from one metabolic reaction (one-reaction library) and two metabolic reactions (two-reaction library)^23^. For relative quantification of a metabolite, the ratio of the average peak value (^12^C-labeled individual sample over ^13^C-labeled counterpart from the pool) in one treatment to that in another treatment to which it was being compared was used to measure fold changes in the level of metabolites between the treatments.

### Statistical analysis

The processed raw data were uploaded to MetaboAnalyst version 5.0 (McGill University, Montreal, Canada)^24^ to analyze expression profiles among the treatments. Data were normalized by median and auto-scaled to make individual features more comparable. Unpaired two-tailed Student’s t-test was used for a binary comparison (univariate analysis) between treatments. Metabolites with a false discovery rate (FDR)-corrected *p*-value (q-value) of less than 0.05 and with a fold change greater than 1.5 (or less than 0.67) were visualized in a volcano plot for binary comparison. Multivariate analysis was carried out using principal components analysis (PCA) and partial least squares-discriminant analysis (PLS-DA) for modeling the differences between the treatments. A heat map was constructed using the relative abundances of select immunity-related metabolites that differed significantly between treatments. Reference metabolic pathways (of *Arabidopsis thaliana*) were obtained from the Kyoto Encyclopedia of Genes and Genomes (KEGG) database (https://www.genome.jp/pathway/ath01100).

### Plant material

CDC Meadow is a commercial field pea cultivar, which is used in this study complying relevant institutional, national, and international guidelines and legislation.

## Results

At the time of sample collection, only PT seedlings showed moderate disease symptoms, while PBBT and BBT seedlings were symptomless. Figure 1 illustrates the effectiveness of this bacterium (PD-S66) in controlling aphanomyces root rot in pea.

### Metabolome analysis

**Figure 2:**
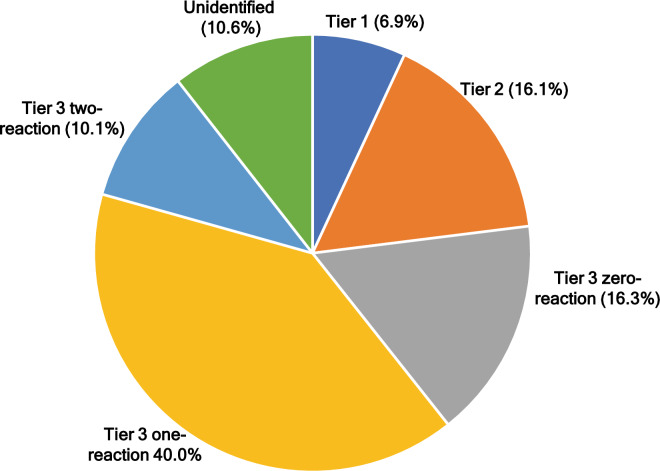
Proportion of metabolites identified in different tiers and unidentified metabolites.

A total of 33 LC–MS dataset were processed for each channel. All labeled metabolites were identified as peak pairs on mass spectra. For bacterium samples, 3490 ± 22 peak pairs were detected, while 3129 ± 38 peak pairs were detected for *A. euteiches* (pathogen) samples. For pea root samples, 5718 ± 26 peak pairs were detected after filtering. The 2-channel analysis detected a total of 5835 unique peak pairs of putatively identified and unidentified metabolites (Suppl. Table 1). Among those unique peak pairs, 5249 pairs (89.4%) were positively identified or putatively matched using a three-tier approach (Fig. 2; Suppl. Table 1). Out of those, 403 peak pairs were positively identified in tier 1. After identification in tier 1, the remaining peak pairs were compared with LI Library and a total of 940 peak pairs were identified with high confidence (Tier 2). The remaining peak pairs were searched against MCID library and 952, 2335, and 589 were matched with the zero-, one- and two-reaction libraries, respectively, in tier 3 (Fig. 2).

### Comparison of metabolites between treatments

**Figure 3:**
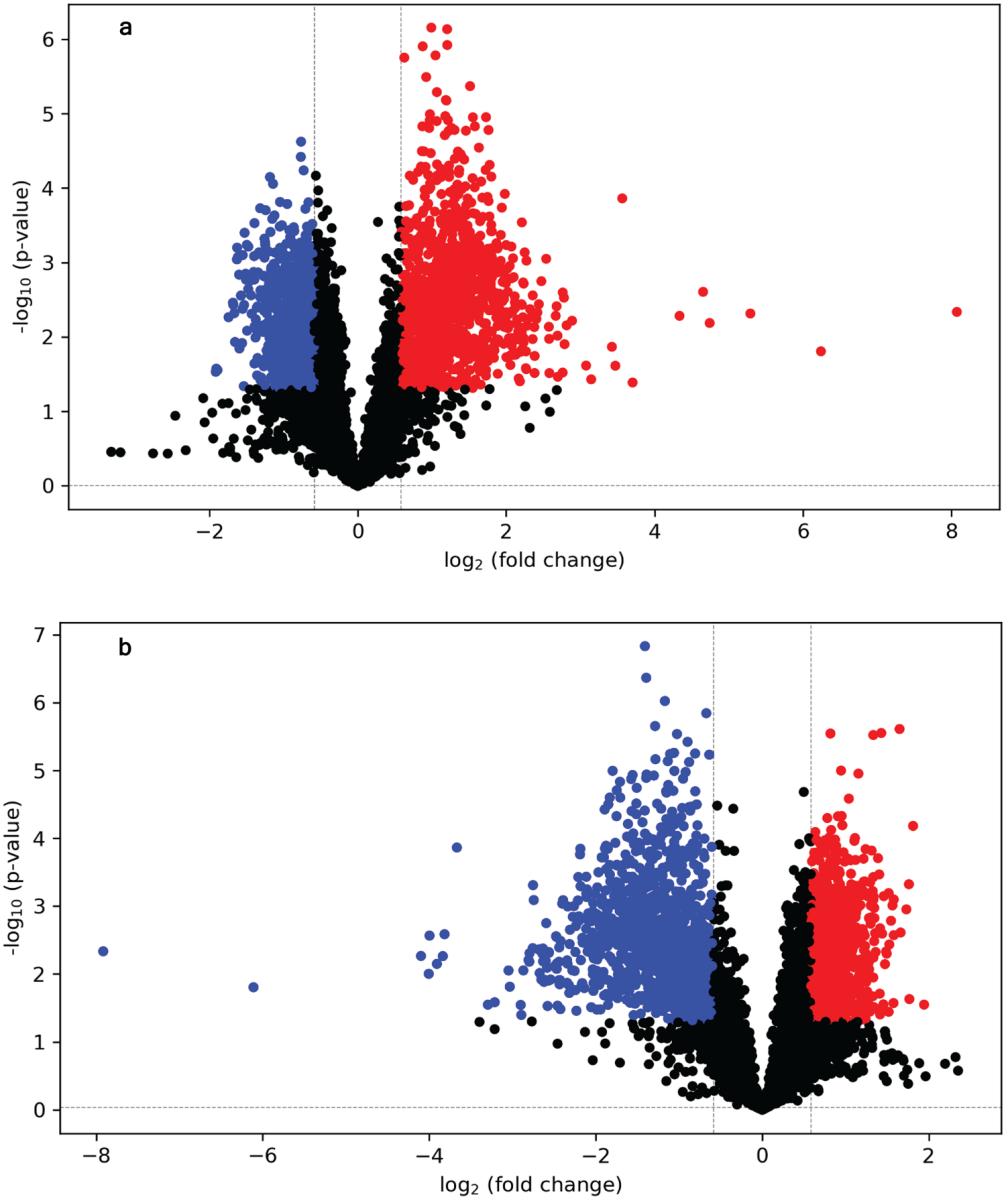
Volcano plots showing the comparison of metabolites levels in roots between (**a**) pea treated with *A. eutieches* (PT) and plants without pathogen or bacterial treatment (control) or (**b**) pea treated with *A. eutieches* + biocontrol bacterium (PBBT) and PT. Metabolites with a false discovery rate (FDR)-corrected *p*-value (q-value) of less than 0.05 and with a fold change greater than 1.5 (or less than 0.67) were considered significantly different. Up-regulation, down-regulation, and non-significance are denoted as red, blue, and black circles, respectively.

In univariate analysis, a total of 1112 and 1094 metabolites were significantly up- and down-regulated, respectively, in PT relative to control (Fig. 3a). Among those, 197 peak pairs were positively identified in tier 1, 329 peak pairs were putatively identified with high-confidence in tier 2, and 1450 peak pairs could be putatively identified in tier 3 (Suppl. Table 2). The metabolites associated with pathways potentially relevant to plant responses to *A. euteiches* and/or plant growth and development, identified in tier 1 and tier 2, are shown in Suppl. Table 3. A total of 1114 and 1092 metabolites were significantly up- and down-regulated, respectively, in PBBT in relation to PT (Fig. 3b). Among those, 205 peak pairs were positively identified in tier 1, and 298 and 1447 could be putatively identified in tiers 2 and tier 3, respectively (Suppl. Table 4). The metabolites linked to pathways likely to be relevant to the pea-Aphanomyces-bacterium system, including development and growth of pea, and identified in tier 1 and tier 2 are shown in Suppl. Table 3.

There were no significant (FC > 1.5 or FC < 0.67, *p*-value < 0.05) differences in metabolites levels between control and BBT (Suppl. Fig. 1a; Suppl. Table 3); however, three and one metabolites were up- and down-regulated, respectively, between control and PBBT (Suppl. Fig. 1b; Suppl. Table 3).

**Figure 4:**
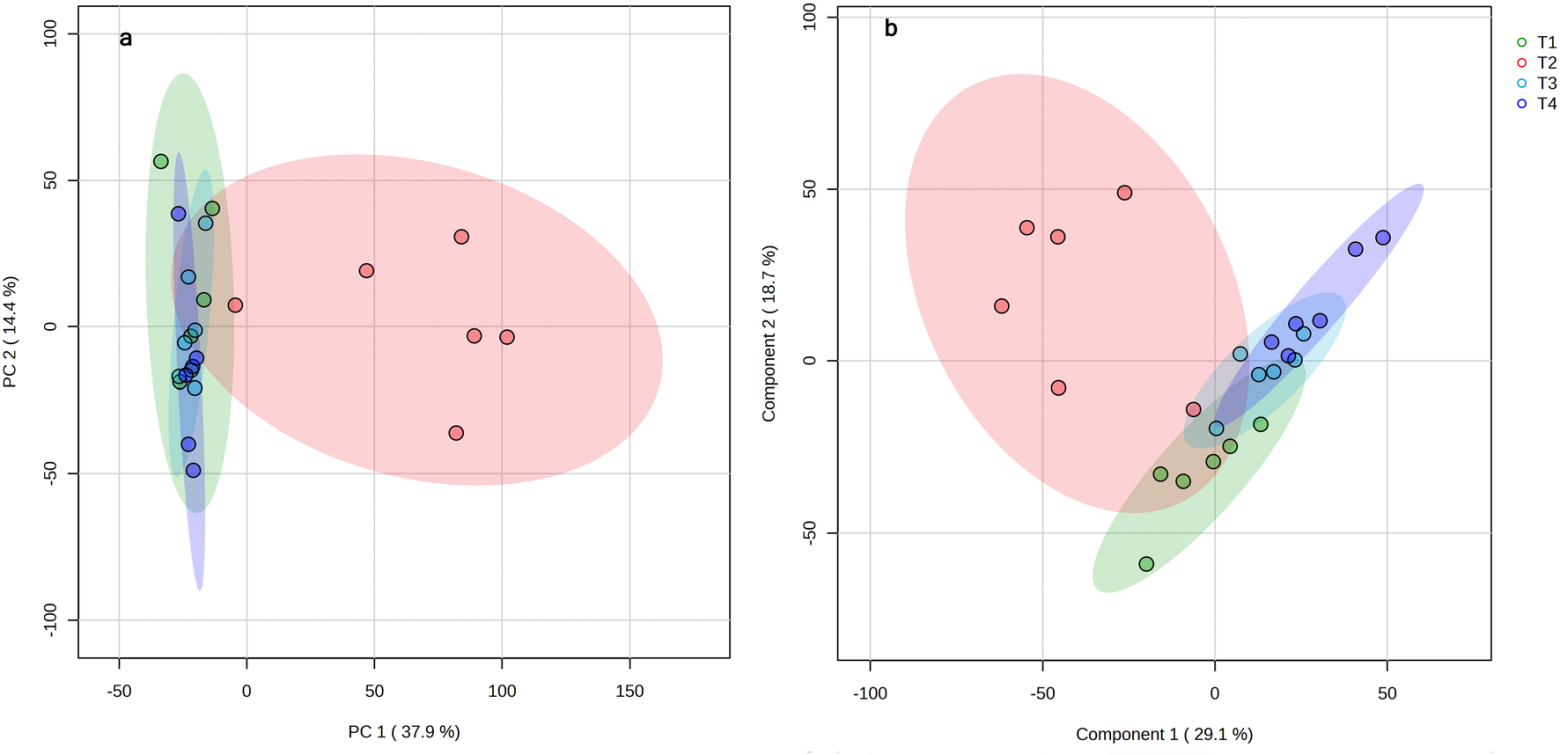
Score plot of (**a**) principal component analysis (PCA) or (**b**) partial least squares-discriminant analysis (PLS-DA) for control (green), PT (red), BBT (sky blue), and PBBT (blue) groups.

In multivariate analysis, samples from control, BBT, and PBBT clustered closely together and were clearly separated from the PT samples in PCA (Fig. 4a). Samples from T2 were more dispersed than those of control, BBT or PBBT. The first two principal components explained 37.9 and 14.4% of the data variance, respectively. In PLS-DA, a supervised multivariate analysis tool, although the trend was similar to PCA, clustering of the samples from control slightly separated from the samples of BBT and PBBT (Fig. 4b). In PLS-DA, first two components explained for 29.1 and 18.7% of the variance, respectively.

Approximately 5000 metabolite features were differentially expressed between the treatments in this study. To clearly visualize the differences, we constructed a heatmap using relative abundance of select metabolites. The relative abundance of almost all the metabolites in PT were significantly different from those in control, BBT, and PBBT (Suppl. Fig. 2).

### Pathway analysis

**Figure 5:**
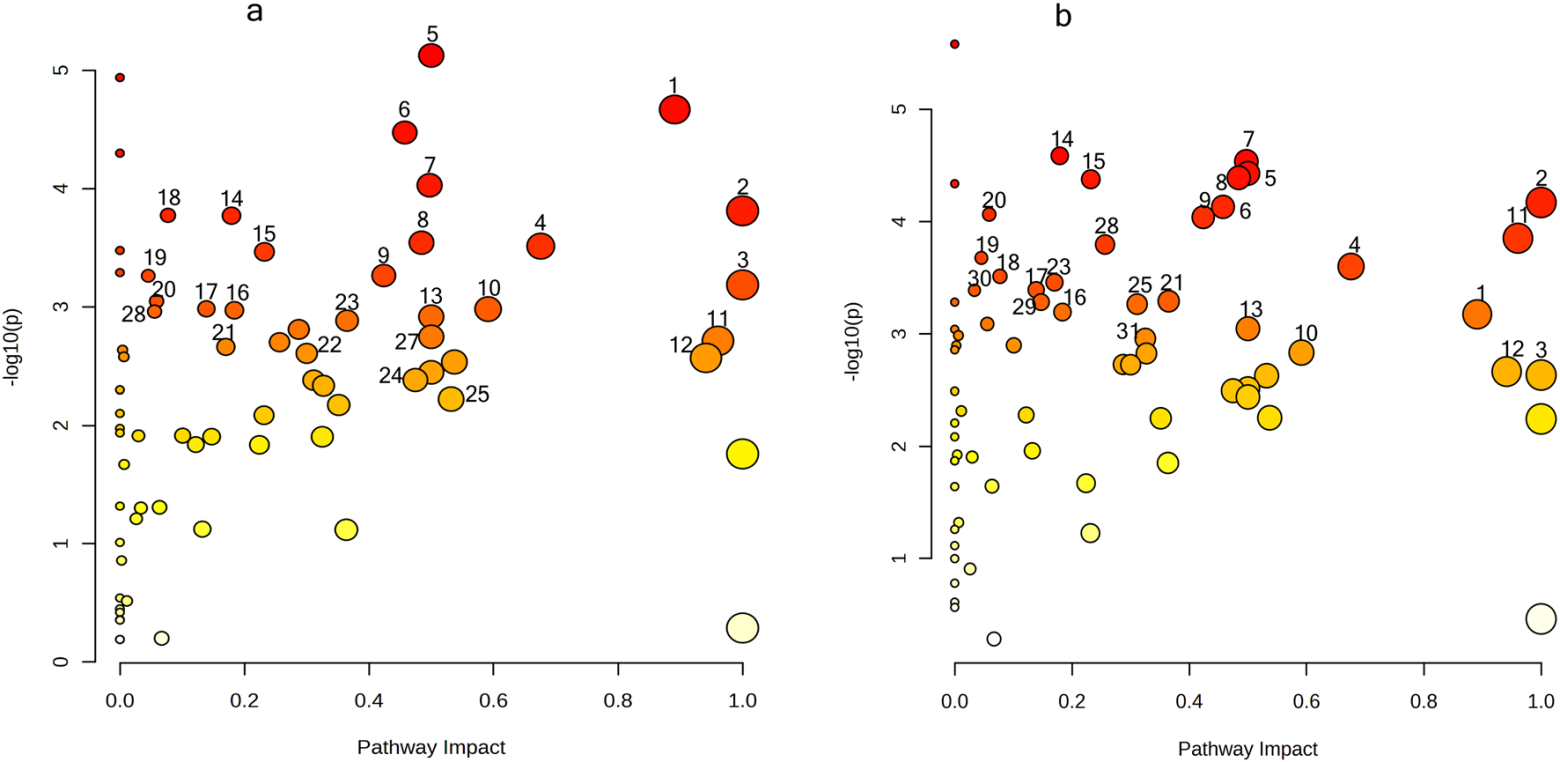
Summary of pathway analysis between (**a**) PT and control or (**b**) PBBT and PT roots. Figure shows all matched pathways arranged according to the *p*-values on the y-axis and pathway impact values on the x-axis; selected pathways include vitamin B6 metabolism (1), linoleic acid metabolism (2), betalain biosynthesis (3), glyoxylate and dicarboxylate metabolism (4), taurine and hypotaurine metabolism (5), alpha-Linolenic acid metabolism (6), cysteine and methionine metabolism (7), glycine, serine and threonine metabolism (8), cyanoamino acid metabolism (9), beta-Alanine metabolism (10), alanine, aspartate and glutamate metabolism (11), phenylalanine metabolism (12), flavone and flavonol biosynthesis (13), purine metabolism (14), sulfur metabolism (15), pyrimidine metabolism (16), tryptophan metabolism (17), glycerophospholipid metabolism (18), flavonoid biosynthesis (19), nitrogen metabolism (20), diterpenoid biosynthesis (21), phenylpropanoid biosynthesis (22), lysine biosynthesis (23), phenylalanine, tyrosine and tryptophan biosynthesis (24), glutathione metabolism (25), and arachidonic acid metabolism (26).

Pathway analysis was conducted using identified metabolites to interpret the metabolic pathways most relevant to pea-*A. euteiches* and pea-*A. euteiches*-bacterium interactions. The analysis revealed that a large number of metabolites significantly related to pea-*A. euteiches* interaction (PT vs control) were associated with 53 metabolic pathways (FDR adjusted *p* < 0.05; Fig. 5a, Suppl. Table 5). Those included cysteine and methionine metabolism; vitamin B6 metabolism; glycine, serine, and threonine metabolism; glyoxylate and dicarboxylate metabolism; alpha-Linolenic acid metabolism; beta-alanine metabolism; alanine, aspartate, and glutamate metabolism; phenylpropanoid biosynthesis; phenylalanine metabolism; flavonoid biosynthesis; flavone and flavonol biosynthesis, and diterpenoid biosynthesis. Metabolites significantly related to pea-*A. euteiches*-bacterium interactions (PBBT vs PT) were associated with 60 metabolic pathways (FDR adjusted *p* < 0.05). Although levels of individual metabolites varied between treatments, associated pathways were similar (Fig. 5b, Suppl. Table 6).

### Changes in metabolites level in PT vs control and PBBT vs PT

As most of the metabolite levels changed significantly between PT and control or between PBBT and PT, we listed some of the metabolites from those two groups with a potential role in plant immunity in Table 1. Because of the roles of the phenylalanine pathway, fatty acids (FAs), and amino acids and their derivatives in plant defense responses, and differences in levels between treatments, we focused on metabolites associated with these pathways. However, level of some other metabolites such as xanthine and dopamine quinone also changed due to pea-*A. euteiches* or pea-*A. euteiches*-bacterial interactions (Table 1). Two of the most important defense hormones, SA and JA were upregulated in PT compared to control or PBBT. Most of the metabolites in phenylalanine pathway and FAs were upregulated in PT compared to control or PBBT. A few metabolites in the phenylpropanoid pathway, such as 5-O-caffeoylshikimic acid, eriodictyol chalcone, kaempferol, and luteolin, were up-regulated in PBBT compared to PT. All the select metabolites in FA biosynthesis, including oleic, linoleic, and alpha-linolenic acids or their derivatives were upregulated in PT compared to control or PBBT. Arachidonic acid, usually found in animals and some microbes^25^, and its derivatives, also accumulated significantly in PT. Arachidonic acid was also present at high levels in the pure *A. eutieches* culture. Some amino acids and derivatives, such as phenylalanine, methionine, arginine, and leucine, were upregulated in PT. However, others, such as asparagine, cysteine, homoserine, and threonine, were upregulated in PBBT. The primary metabolites, in general, did not vary significantly among the treatments. However, two tricarboxylic acid cycle intermediates—malic acid (MA) and oxaloacetic acid—were up-regulated in control and PBBT (Table 1).

**Table 1:** Comparison of select metabolites levels that changed significantly in *A. euteiches*-treated (PT) vs control and *A. euteiches* + bacterium-treated (PBBT) vs PT roots.

Compound	Pathway/panel name	Identification	PT vs control		PBBT vs PT	
			Fold change	*p*-value	Fold change	*p*-value
Salicylic acid	Phytohormone	Tier 1	2.8977	0.0043	0.4739	0.0103
Methyl salicylate	Phytohormone	Tier 2	1.8407	0.0098	0.5563	0.0121
Jasmonic acid	Phytohormone	Tier 1	3.1003	0.0047	0.3519	0.0054
12—Oxo-phytodienoic acid (12-OPDA)	Phytohormone	Tier 2	0.647	0.0473	1.9477	0.0111
1-Aminocyclopropane-1-carboxylic acid	Phytohormone	Tier 2	0.6243	0.0083	1.8513	0.0001
1-Naphthaleneacetic acid	Phytohormone	Tier 2	0.764	ns	1.8684	0.0014
Kinetin	Phytohormone	Tier 2	0.4176	0.0015	2.1843	0.0012
3-Hydroxyphenylacetic acid	Phenylalanine metabolism	Tier 1	2.2266	0.0008	0.3776	0.001
Phenylacetic acid	Phenylalanine metabolism	Tier 1	1.6378	0.008	0.6786	ns
Trans-2,3-Dihydroxycinnamic acid	Phenylalanine metabolism	Tier 2	1.7316	0.0013	0.5482	0.001
4-Hydroxystyrene	Phenylpropanoid biosynthesis	Tier 2	1.9004	0.0052	0.4878	0.004
4-Hydroxycinnamyl aldehyde	Phenylpropanoid biosynthesis	Tier 2	1.8292	0.0037	0.522	0.0032
5-O-Caffeoylshikimic acid	Phenylpropanoid biosynthesis	Tier 2	0.8065	ns	1.5381	0.0306
Cinnamic Acid/Trans-Cinnamic Acid	Phenylpropanoid biosynthesis	Tier 1	4.1587	0.0238	0.2772	0.0284
Benzoic Acid	Phenylalanine metabolism/Benzoate degradation	Tier 1	2.41	0.0028	0.4681	0.0042
4-Hydroxybenzoic acid	Phenylalanine metabolism/Benzoate degradation	Tier 1	1.5798	0.0081	0.5622	0.0039
3-Hydroxybenzoic acid	Phenylalanine metabolism/Benzoate degradation	Tier 1	1.5126	0.0064	0.5854	0.0018
Chrysoeriol	Flavone and flavonol biosynthesis	Tier 2	2.5131	0.001	0.3653	0.001
Eriodictyol chalcone	Flavonoid biosynthesis	Tier 2	0.514	ns	2.0513	0.007
Kaempferol	Flavonoid biosynthesis	Tier 2	0.4897	0.0037	2.0748	0.0019
Luteolin	Flavonoid biosynthesis	Tier 2	0.5984	0.0385	1.7419	0.0208
Palustric acid	Diterpenoid biosynthesis	Tier 2	15.6405	0.0079	0.0753	0.0083
Pisiferic acid	Diterpenoid biosynthesis	Tier 2	2.7191	0.0023	0.3839	0.0023
9β-pimara-7,15-dien-19-oic acid	Diterpenoid biosynthesis	Tier 2	7.8840	0.006	0.1689	0.0069
18-Oxooleic acid	Cutin, suberine, and wax biosynthesis	Tier 2	2.4383	0.000	0.4739	0.0001
Hexadecanedioic acid	Cutin, suberine, and wax biosynthesis	Tier 1	1.6347	0.0028	0.7817	ns
9,10-Dihydroxystearic acid	Cutin, suberine, and wax biosynthesis	Tier 2	2.8158	0.0084	0.5074	0.0241
3,4-Dihydroxy-l-phenylalanine	Betalain/isoquinoline alkaloid biosynthesis	Tier 2	0.5944	0.0191	1.345	ns
Dopamine quinone	Betalain biosynthesis	Tier 2	2.1006	0.003	0.4233	0.0017
2,4-Dichlorobenzoic acid	Fluorobenzoate degradation	Tier 2	0.7566	ns	1.632	0.0191
3-Hydroxyanthranilic acid	Aminobenzoate degradation	Tier 2	3.5876	0.0128	0.2099	0.009
Vanillyl alcohol	Aminobenzoate degradation	Tier 2	3.1537	0.044	0.2409	0.031
4-Hydroxy-3-methylbenzoic acid	Toluene degradation	Tier 2	1.8407	0.0098	0.5564	0.012
Oleic acid	Long-chain fatty acid	Tier 1	1.9806	0.003	0.703	ns
Octadec-9-ene-1,18-dioic-acid	Oleic acid metabolism/cutin, suberine, and wax biosynthesis	Tier 2	2.6259	0.001	0.424	0.0013
Linoleic acid	Long-chain fatty acid	Tier 1	2.9987	0.0008	0.3395	0.0013
13-Oxo-octadecadienoic acid (13-OxoODE)	Linoleic acid metabolism	Tier 2	1.9185	0.003	0.6274	0.024
(8Z,11Z,14Z)-Icosatrienoic acid	Linoleic acid metabolism	Tier 2	20.1837	0.0052	0.0583	0.0054
Crepenynic acid	Linoleic acid metabolism	Tier 2	2.5701	0.0024	0.5208	0.0054
Alpha-Linolenic acid	Long-chain fatty acid	Tier 1	3.5076	0.001	0.3338	0.002
Colnelenic acid	Alpha-linolenic acid metabolism	Tier 2	2.1529	0.0005	0.4596	0.0005
17-Hydroxylinolenic acid	Alpha-linolenic acid metabolism	Tier 2	2.3363	0.0038	0.5298	0.0095
13(*S*)-hydroperoxy-9(*Z*),11(*E*),15(*Z*)-octadecatrienoic acid [13(S)-HPOT]	Alpha-linolenic acid metabolism	Tier 2	2.3652	0.0004	0.4376	0.0003
9(*S*)-hydroxy-10,12,15-octadecatrienoic acid [9(S)-HOT]	Alpha-linolenic acid metabolism	Tier 2	2.7885	0.002	0.4134	0.002
Heptadecatrienoic acid	Alpha-linolenic acid metabolism	Tier 2	2.8597	0.008	0.5031	0.024
9-Oxononanoic acid	Alpha-linolenic acid metabolism	Tier 2	1.6517	0.0096	0.6073	0.01
Heptadecatrienoic acid	Alpha-linolenic acid metabolism	Tier 2	2.8596	0.0081	0.5031	0.024
Icosadienoic acid	Biosynthesis of unsaturated fatty acids	Tier 2	2.815	0.0004	0.3966	0.0008
Stearidonic acid	Alpha-linolenic acid metabolism	Tier 2	2.286	0.0003	0.5299	0.0011
Azelaic Acid	Fatty acids and conjugates	Tier 1	2.599	0.0088	0.3169	0.0059
Arachidonic acid	Long-chain fatty acid	Tier 2	269.0955	0.0046	0.0041	0.0046
15-oxo-5Z,8Z,11Z,13E-eicosatetraenoic acid (15-OxoETE)	Arachidonic acid metabolism	Tier 2	2.8136	0.0024	0.4223	0.004
15-Hydroxy-11,12-epoxyeicosatrienoic acid (15H-11,12-EETA)	Arachidonic acid metabolism	Tier 2	6.4508	0.0082	0.243	0.012
5,6-Epoxytetraene	Arachidonic acid metabolism	Tier 2	2.5257	0.0007	0.4483	0.0008
Asparagine	Alanine, aspartate, and glutamate metabolism	Tier 1	0.5141	0.008	2.1739	0.001
Alanine	Amino Acids & Derivatives	Tier 1	1.8042	0.010	0.6170	0.018
Arginine	Amino Acids & Derivatives	Tier 1	1.9602	0.004	0.6372	0.014
Aspartic acid	Alanine, aspartate, and glutamate metabolism	Tier 1	1.6675	0.0117	0.6483	0.020
Cysteine	Amino Acids & Derivatives	Tier 1	0.6522	0.047	1.6388	0.0354
Glutamine	Arginine biosynthesis	Tier 1	0.8541	ns	1.7183	0.029
Glutamic Acid	Amino Acids & Derivatives	Tier 1	1.7164	0.0183	0.5583	0.014
Homoserine	Glycine, serine, and threonine metabolism	Tier 1	0.5913	0.0223	2.0652	0.0014
Leucine	Amino Acids & Derivatives	Tier 1	1.9900	0.008	0.5721	0.015
Methionine	Amino Acids & Derivatives	Tier 1	3.3892	0.015	0.3306	0.017
Phenylalanine	Amino Acids & Derivatives	Tier 1	1.8402	0.0035	0.5281	0.0034
Threonine	Valine, leucine, and isoleucine biosynthesis	Tier 1	0.7098	ns	1.5967	0.0015
Chorismic acid	Phenylalanine, tyrosine and tryptophan biosynthesis	Tier 2	0.5693	0.0297	1.3349	ns
Pipecolic acid	Lysine degradation	Tier 1	1.8935	0.0285	0.5567	0.0358
N-Nitrosoproline	Amino Acids & Derivatives	Tier 2	0.6168	0.0126	1.7068	0.0063
Ophthalmic acid	Amino Acids & Derivatives	Tier 2	4.1317	0.001	0.1892	0.001
5-Hydroxyindoleacetic acid	Tryptophan metabolism	Tier 1	0.6619	0.0137	1.5534	0.007
4-Hydroxyphenylethanol	Tyrosine metabolism	Tier 2	2.1864	0.0371	0.355	0.019
Imidazoleacetic acid	Histidine metabolism	Tier 1	0.4802	0.0049	1.6949	0.0282
Carnitine	Lysine degradation	Tier 1	3.3771	0.002	0.2325	0.002
5-Hydroxyectoine	Amino Acids & Derivatives	Tier 2	2.3815	0.0018	0.3569	0.001
p-Coumaroylputrescine	Arginine and proline metabolism	Tier 2	1.8683	0.0001	0.4771	0.00001
Succinic Semialdehyde	Alanine, aspartate, and glutamate metabolism	Tier 1	1.9935	0.024	0.3874	0.010
2-Methyl-3-hydroxy-5-formylpyridine-4-carboxylic acid	Vitamin B6 metabolism	Tier 2	0.5366	0.0161	1.5348	ns
4-Pyridoxic acid	Vitamin B6 metabolism	Tier 2	0.5097	0.0011	1.6563	0.0091
4-Pyridoxolactone	Vitamin B6 metabolism	Tier 2	1.7752	0.0018	0.5088	0.0009
Isopyridoxal	Vitamin B6 metabolism	Tier 2	0.5807	0.003	1.7458	0.0029
Pyridoxamine	Vitamin B6 metabolism	Tier 2	0.5578	0.0015	1.4341	ns
Pyridoxamine phosphate	Vitamin B6 metabolism	Tier 2	0.5011	0.0338	2.0041	0.0084
Xanthine	Caffeine metabolism	Tier 1	4.6943	0.0119	0.1569	0.010
Malic acid	Tricarboxylic acid cycle	Tier 1	0.4746	0.005	1.9214	0.018
Oxaloacetic acid	Tricarboxylic acid cycle	Tier 2	0.4835	0.0108	2.5592	0.002
Threonic acid	Ascorbate and aldarate metabolism	Tier 1	2.8424	0.016	0.2528	0.009

## Discussion

The importance of the rhizosphere and/or soil microbiome in shaping plant performance is widely recognized^17^. Plants also shape the rhizosphere and soil microbiome by secreting a diverse set of metabolites as root exudates. We explored plant interactions with a pathogen, a beneficial microbe, and both types of microorganisms, using untargeted metabolomics. Both univariate and multivariate analyses clearly demonstrated the differences in metabolites levels between PT and other treatments (control, BBT, and PBBT). Differences in metabolites levels that were exclusively observed between PT and control, and PBBT and PT (Fig. 3) suggest that metabolites levels in pea roots changed due to *A. euteiches* infection or suppression of *A. euteiches* by the bacterium. Absence of differences between PBBT and control indicate that bacterium-mediated suppression of *A. euteiches* likely resulted in infection-mediated changes in metabolites levels not occurring (Suppl. Fig. 1b). Selected metabolites that were significantly upregulated or downregulated between PT vs control and PBBT vs PT are presented in Table 1 and discussed below in terms of biological relevance.

### Defense hormones

SA, JA, and ET are the classic plant immunity hormones; their importance in the plant defense signaling network is well established^26^. Extensive studies in model plant *A. thaliana* demonstrated that SA signaling generally confers resistance against biotrophic and hemibiotrophic pathogens, whereas JA/ET signaling is mostly associated with resistance to necrotrophic pathogens^27^. SA mediates local resistance in the infected region as well as systemic resistance at the whole plant level. Pathogen infections usually lead to a rapid increase in SA levels^28^. In this study, SA levels significantly increased in PT roots compared to the control as well as PBBT roots (Table 1). In addition to SA, more mobile signals are required for systemic acquired resistance (SAR) induction. Several signals, including azelaic acid, pipecolic acid, methyl salicylate, glycerol-3-phosphate, and dehydroabietinal, have been identified in plants^29^. Up-regulation of methyl salicylate, azelaic acid, and pipecolic acid in PT compared to control or PBBT suggests a role of these metabolites in SAR establishment in pea in response to *A. euteiches* infection. Although JA plays a key role in modulating defense against necrotrophic pathogens, it has also been shown to mediate defense against some biotrophic and hemibiotrophic pathogens^30^. For example, JA reduces plant susceptibility to bacterial (*Pseudomonas syringe* and *Xanthomonas campestris*), fungal (*Verticillium dahliae* and *Fusarium oxysporum* f. sp. *lycopersici*), and oomycete (*Phytophthora infestans*) pathogens^31^. Consistently, we found significantly higher levels of JA in *A. euteiches*-treated pea roots, suggesting a role of this hormone in defense against this hemibiotrophic pathogen in pea.

The antagonistic relationship of SA and JA is well established, particularly when plants are exposed to pathogens^28,32^. For example, Shim et al.^32^ demonstrated an antagonistic interaction between SA and JA in Arabidopsis when challenged with a biotrophic pathogen (*Pseudomonas syringe* pv. *tomato* (*Pto*) DC3000) or a necrotrophic pathogen (*Alternaria brassicicola*). By studying the *npr1-1* mutant and *NahG* transgenic plants, Spoel et al.^33^ also demonstrated that the reduced SA accumulation results in a dramatic increase in JA levels in response to *Pto* DC3000 infection. Despite well documented antagonism, synergistic interactions between these two defense hormones are not uncommon. For example, the synergistic effect of SA and JA-mediated defenses against the hemibiotrophic pathogens *Magnaporthe oryzae* and *Xanthomonas oryzae* pv. o*ryzae* has been observed in rice^34^. Liu et al.^30^ also demonstrated that both SA and JA accumulated to high levels in response to *Ps* pv. *maculicola* ES4326 infection in Arabidopsis during effector triggered immunity-induction. Simultaneous accumulation of both SA and JA improves plants resistance against both biotrophic and necrotrophic pathogens or against hemibiotrophic pathogens such as *A. euteiches*. As *A. euteiches* switches to a necrotrophic life cycle, JA-mediated immunity may protect pea from this pathogen. This synergistic interplay between SA and JA offers a possible explanation of the higher levels of both SA and JA in pea roots infected by *A. euteiches*.

12-Oxo-phytodienoic acid (12-OPDA) is a precursor of JA, but the level of this metabolite was higher in PBBT or control than PT. Although this phenomenon seems contradictory, it is consistent with the fact that 12-OPDA can act as an independent signaling molecule^35,36^. For example, *OPR3*-silenced tomato mutants contain significantly lower levels of 12-OPDA and downstream JA derivatives; however, treatment with 12-OPDA, not JA, significantly contributed to restoring plant basal resistance against *Botrytis cinerea*^37^. By using ISR-positive and -negative mutants of maize (*Zea mays*) and inoculation with the beneficial fungus *Trichoderma virens* (Tv), Wang et al.^36^ identified 12-OPDA as an important ISR signal against *Colletotrichum graminicola,* a hemibiotroph. 12-OPDA has antifungal activity and has been reported to inhibit growth of several fungal pathogens^38^. This suggests that enhanced 12-OPDA levels in PBBT might negatively affect the growth/colonization of *A. euteiches* in pea roots.

1-Aminocyclopropane-1-carboxylic acid (ACC) was upregulated in PBBT compared to PT. In plants, ACC is converted to ethylene by ACC oxidase (ACO). In addition to being crucial for ethylene biosynthesis, recent evidence suggests that ACC can also act as a signaling molecule, independent of its conversion to ethylene^39^. For example, ACC is a potential negative regulator of virulence in *V. dahlia* and a positive regulator of defense in tomato and eggplant^40^. Application of ACC also resulted in enhanced resistance against *P. syringae* pv. *Tomato* in *Arabidopsis*^41^. Thus, increased ACC levels in PBBT may be associated with bacterial suppression or reduced virulence of *A. euteiches* in pea. JA and ET are important in the regulation of the SA-independent systemic immunity provided by beneficial soil-borne microbes. Rhizobacteria-mediated ISR was shown to be effective against attackers that are sensitive to JA/ET-dependent defenses, including necrotrophic pathogens^8^. The upregulation of ACC might be an indication of SA-independent, but ET dependent, induction of ISR mediated by the bacterium in PBBT.

### Fatty acids (FAs) and derivatives

FAs and FA-derived metabolites are the major source of reserve energy and essential components of cellular membranes in living organisms. Palmitic (16:0), stearic (18:0), oleic (18:1_Δ9_), linoleic (18:2_Δ9,12_), and linolenic (18:3_Δ9,12,15_) acids are common FAs found in plant lipids^42^. The unsaturated FAs 18:1, 18:2, and 18:3 and their derivatives act as signaling molecules and play a crucial role in plant–microbe interactions^25^. These FAs can act directly as free FAs or as oxylipins, a huge and diverse family of oxygenated polyunsaturated fatty acids (PUFAs) derivatives. The PUFAs (such as 18:3) generally induce protein kinase C-mediated activation of NADPH oxidase, resulting in the production of reactive oxygen species (ROS) and subsequent defense responses during *R* gene–mediated resistance in plants^43,44^. In this study, higher levels of 18:2 and 18:3 as well as their derivatives in PT compared to control or PBBT (Table 1) may be associated with SAR-mediated pea defense responses against *A. euteiches*. Consistently, increased levels of 18:2 and 18:3 resulted in higher resistance to *Colletotrichum gloeosporioides* in avocado^45^ and *Pseudomonas syringae* in tomato^43^. Similarly, *Arabidopsis fad7 fad8* mutant, which is defective in 18:3 syntheses (defective in the desaturation of 18:2 to 18:3) in the chloroplastic membranes shows enhanced susceptibility to *P. syringae*^43^. Elevated level of PUFAs is associated with biocontrol agent-induced disease resistance. For example, Rhizobacteria-induced enhanced resistance to *Botrytis cinerea* is linked to the accumulation of 18:2 and 18:3 FAs in bean^46^. In contrast, PUFAs and their derivatives are also important for sporulation, sexual structure development, and host colonization in some mycotoxic fungi such as *Aspergillus* spp.^47,48^, and thereby contribute to pathogen (*A. eutieches*) fitness.

In our study, α-linolenic acid derivatives 9(*S*)-hydroxy-10,12,15-octadecatrienoic acid [9(S)-HOT], 13(*S*)-hydroperoxy-9(*Z*),11(*E*),15(*Z*)-octadecatrienoic acid [13(S)-HPOT], and colnelenic acid increased in response to *A. euteiches* infection. 9-HOT enhances brassinosteroid signaling and cell wall-based defense responses, and induces ROS production^49^. 13-HPOT participates in a lipid-based signaling system initiated by insect and pathogen attack in plants. It is a precursor of a number of biologically active oxylipins, including JA, which plays a critical role in plant responses to pathogens, wounding, and herbivory^50^. Previously, Göbel et al.^51^ reported increased levels of 9-LOX-derived 9,10,11- and 9,12,13-trihydroxy derivatives of linolenic acid (LnA), divinyl ethers colnelenic acid (CnA) and colneleic acid (CA) in potato leaves in response to *Phytophthora infestans* infection. The oxylipins whose levels were elevated in this study in pathogen–infected pea likely contributed to defense responses. In addition, induction of oxylipins in pea roots is likely to have a negative impact on *A. euteiches* growth because of their antimicrobial activity^38,52^. An antagonistic relationship between ACC and 9-HOT was found previously^53^. Consistently, we found lower 9-HOT abundance and higher ACC abundance in PBBT compared to PT (Table 1).

In contrast, reduction of 18:1 level led to an increase in endogenous nitric oxide (NO) levels, which triggers transcriptional upregulation of a number of diverse *R* genes in an SA-independent manner and boost plant defense during pathogen infection^54^. Reduced abundance of 18:1 and derivatives (18-Oxooleic and octadec-9-ene-1,18-dioic-acids) in PBBT compared to PT (Table 1) might enhanced plant defense responses and thus, contributed to suppression of *A. euteiches* by the bacterium. Conversely, expression of the yeast Δ-9 desaturase gene in eggplant resulted in increased levels of 16:1, 18:1, and 16:3 fatty acids that enhanced *Verticillium dahliae* resistance^55^. The 18:1 and its derivatives are also precursors of cutin and suberin biosynthesis, two compounds that provide protection against pathogens^56^. Therefore, it is also possible that increased levels of 18:1 and derivatives in PT act as signaling molecule to boost cutin and suberin biosynthesis to provide protection against *A. euteiches* in pea. Levels of 18:1 or its derivatives in PT and PBBT present a complex scenario, suggesting that 18:1 level might be adjusted in at the spatio-temporal level to optimize defense responses. Some FAs such as eicosapolyenoic acids (EP), arachidonic acid (AA, 20:4) and eicosapentaenoic acid (20:5) are not commonly found in plants, but these are common in plant pathogenic oomycetes^25^. These FAs are released into plant tissue from pathogen spores during infection and can function as signaling molecules and trigger FA-mediated defense responses^57,58^. In addition, the presence of foreign FAs may perturb plant oxylipin metabolism, altering the course of 18:2 and 18:3 peroxidative metabolism and elicit a plant response to the invading pathogen. For example, induction of SAR and subsequent disease reduction has been observed in AA-treated potato infected with *Phytophthora infestans*^59^. It is possible that the presence of AA and its derivatives in PT (Table 1) acted as signaling molecules to initiate defense responses against *A.* euteiches^25^. We also found a significant amount of AA in *A. euteiches* (data not shown), consistent with the findings that oomycetes contain AA^25,57^. AA detected in PT was likely produced by *A. euteiches*. However, like control, PBBT contained negligible amount of AA. It is hypothetically possible that bacterium-mediated suppression of *A. euteiches* meant that *A. euteiches* abundance in the roots was insufficient to produce significant levels of AA.

### Phenylalanine-derivatives

Phenylalanine, an aromatic amino acid, is a common precursor to a large array of phenolic compounds, including flavonoids, isoflavonoids, condensed tannins, lignans, lignin, and phenylpropanoid/benzenoid volatiles^60^. Among other functions, these metabolites are involved in plant defense and signaling^61,62^. Several phenolic compounds were upregulated in PT, while levels of few were also increased in PBBT (Table 1). For example, levels of trans-2,3-dihydroxycinnamic acid and 4-hydroxycinnamyl aldehyde, associated with lignin and lignan biosynthesis, increased in PT. Lignin is a major structural component of secondary cell walls in vascular plants and involved in a wide range of functions, including physical barriers against pathogen infection^63^. Hydroxybenzoic acids (C6–C1) are phenolic acids with diverse biological functions in plants, including plant–microbe symbiosis, allelopathic activities, and resistance to pathogen attack^64^. The best known benzoic acid (BA), SA (2-hydroxybenzoic acid) is a key signaling molecule, which activates plant defense against a wide variety of pathogens, and is essential for both local and SAR^65^. In addition to SA, the levels of several BAs such as 3-hydroxybenzoic acid, 4-hydroxybenzoic acid, and 3-hydroxyanthranilic acid were higher in PT than in control or PBBT. 4-hydroxybenzoic acid can suppress the hyphal growth of *A. euteiches* in vitro^66^, implying that increased level of this metabolite may boost pea resistance to the pathogen. In contrast, more 2,4-dichlorobenzoic acid accumulated in PBBT than in PT (Table 1). Overall, increased levels of BA-derivatives in response to *A. euteiches* infection in pea is consistent with their role in plant responses to biotic stress.

Flavonoids participate in a large number of physiological and biochemical processes such as photosynthesis, respiration, growth and development, and plant defense against various stresses, including pathogenic microbes^67^. For example, treating spikes with exogenous kaempferide and apigenin increased wheat (*Triticum aestivum* L.) resistance to fusarium head blight caused by *Fusarium graminearum* Schwabe^68^. Apigenin concentration also increased in diseased lentil root tissues in response to *A. euteiches* infection^66^*.* Eriodictyol chalcone, kaempferol, and luteolin were upregulated in PBBT compared to PT, while another flavonoid, chrysoeriol, was upregulated in PT compared to control or PBBT (Table 1). Luteolin has antioxidant activity and inhibits ROS-induced damage of lipids, DNA, and protein. It also exhibited toxicity to spores of *Colletotrichum sublineola*, the causal agent of anthracnose in sorghum^69^. In contrast, the reduced accumulation of flavonoids can enhance susceptibility of some plants to pathogen infection^70^. Nazari et al.^71^ also demonstrated that biocontrol PGPR *Bacillus subtilis* can enhance flavonoid accumulation in plant tissues and subsequent contribution to pathogen suppression, which is consistent with our study. These findings suggest that some flavonoids could play a role in bacterial-mediated suppression of *A. euteiches*, while others may contribute to *A. euteiches* resistance in pea. This seems plausible, given the great diversity of plant flavonoids.

Diterpenoids are a chemically and functionally diverse group of 20-carbon terpenoids. Their broad spectrum of functions include defense against pathogens, herbivory, and weeds^72^. These compounds occur at low basal concentrations in plants. However, their levels can be increased in response to exogenous elicitors such as pathogen infection. For example, the diterpenoid dolabralexin was up-regulated in maize roots in response to *Fusarium verticillioides* and *F. graminearum* infection, and epoxydolabranol significantly inhibited the growth of both pathogens in vitro^73^. Accumulation of diterpenoid metabolites, such as palustric, pisiferic, and 9β-pimara-7,15-dien-19-oic acids, in pea roots in response to *A. euteiches* infection (Table 1) is consistent with their role in defense against pathogens. Precursors to, or intermediates in, the cutin, suberine, and wax biosynthesis pathways (such as hexadecanedioic and 9,10-dihydroxystearic acids) were also upregulated in PT. In addition to acting as a physical barrier between plants and their environment, wax and its breakdown products also serve as signaling molecules in response to pathogen attack^74^. Our results suggest these compounds have a role in pea resistance to aphanomyces root rot.

### Amino acid and derivatives

In addition to protein biosynthesis and serving as building blocks for other biosynthesis pathways, amino acids play crucial roles in signaling processes as well as in plant responses to biotic and abiotic stresses^75^. Levels of several amino acids, including alanine, arginine, leucine, methionine, and phenylalanine, were increased in PT compared to control or PBBT (Table 1). Arginine is involved in the biosynthesis of polyamines and NO in higher plants. NO acts as a signaling molecule in activating defense responses against pathogens^76^. It is likely that increased arginine levels in PT are associated with NO production and thus plays a role in enhancing pea defense responses against *A. euteiches*. Methionine (Met) is an essential sulfur-containing amino acid, important in diverse biological processes, including protein translation, biosynthesis of the plant defense hormone ethylene^77^, or DNA methylation. For example, ethylene levels in rice seedlings increased due to exogenous Met application, which resulted in enhanced basal blast (caused by *Magnaporthe* oryzae) resistance^78^. Significant increase of Met in *A. euteiches*-treated pea roots (3.39-fold) suggest a role of this amino acid against aphanomyces root rot on pea, possibly through synthesis of defense-related metabolites. Compounds derivded from phenylalanine function in plant defense or as signaling molecules. In this study, *A. euteiches*-infection enhanced phenylalanine level significantly in pea roots. Yoo et al.^62^ also found significantly increased level of phenylalanine in *Pseudomonas syringae* pv. *maculicola* (*Psm*) ES4326 infected Arabidopsis. These authors demonstrated that phenylalanine has a role in conferring effector-triggered immunity (ETI) in plants. This is also consistent with the upregulation of some metabolites within the phenylpropanoid pathway in this study since phenylalanine is a precursor of phenylpropanoids biosynthesis. Leucine-rich repeat (LRR) are typically 24 amino acid motif of leucine-rich consensus sequences which can occur multiple times in a single protein^79^ and regulate the activation of many plant defense genes. Increased leucine in PT may indicate upregulation of LRR proteins to protect pea plants from *A. euteiches* infection. Alanine is associated with growth promotion of *Fusarium oxysporum* and *F. solani* in peanut^80^. High alanine levels in PT might be related to *A. euteiches* colonization on pea.

Amino acids such as asparagine, cysteine, glutamine, homoserine, and threonine levels were increased in PBBT compared to PT (Table 1). In many higher plants, asparagine (ASN) and glutamine (GLN) are central intermediates in nitrogen metabolism and play a major role in nitrogen transport. The accumulation of ASN can be modulated during stress as part of the nitrogen remobilisation process^81^. Under biotic stress, this remobilisation may deprive pathogens of nitrogen^82^. Reduced ASN in PT roots compared to PBBT or control roots suggests that nitrogen remobilization may be part of the pea response to aphanomyces root rot. The responses of ASN and GLN to plant pathogen infection appears to vary between host–pathogen combinations. Hwang et al.^82^ found that GLN levels did not change and ASN levels increased slightly in response to one foliar pathogen, but not to another. However, Pérez-García et al.^83^ documented an increase in both ASN and GLN levels in response to infection. It is possible that differences in experimental systems resulted in contrasting outcomes. ASN may also have been broken down by asparaginase in the infected roots of pea before analysis in our study. Although the amount of GLN did not differ between PT and control, PBBT had significantly more GLN than PT. Thus, bacterial inoculation is likely to be responsible for elevated GLN levels. Cysteine is a precursor of many essential biomolecules, including vitamins, cofactors, antioxidants, and defense compounds. Multicellular organisms, including plants, produce small cysteine-rich antimicrobial peptides that provide resistance to a broad spectrum of plant pathogens^84^. The higher levels of cysteine in PBBT or control than PT suggests a role of this sulfur-donating amino acid in *A. euteiches* resistance in pea.

### Primary metabolites

MA and oxaloacetic acid levels were significantly greater in PBBT and control compared to PT. MA is involved in plant defense mechanisms, including ISR and ethylene metabolism^85^. MA is also associated with signaling and recruiting beneficial rhizobacterium *Bacillus subtilis* FB17 in Arabidopsis roots^86^. MA levels increased in roots of rice plants inoculated with *Bacillus subtilis* RR4^87^. Bacterial inoculation likely boosted primary metabolism as well as growth and development in pea.

### Metabolites important to plant immunity and oxidative stress tolerance

In addition to acting as a coenzyme in various biochemical reactions^88^, vitamin B6 (VB6) possesses antioxidant activity and plays important roles in cellular antioxidant defense regulation^89,90^. Involvement of VB6 in plant defense responses against biotic stresses have also been demonstrated recently. It is important in regulating defense response against *B. cinerea* in tomato^91^. Samsatly et al.^92^ found that the Arabidopsis mutant *pdx1.3*, compromised in VB6 biosynthesis, is more susceptible to *Rhizoctonia solani* compared to the wild type. Both groups concluded that defense responses of VB6 to the pathogens were regulated by the modulation of cellular antioxidant capacity. In our study, five VB6 metabolites (2-Methyl-3-hydroxy-5-formylpyridine-4-carboxylic acid, 4-Pyridoxic acid, Isopyridoxal, Pyridoxamine, Pyridoxamine phosphate) levels were lower in PT compared to control, and three metabolites (4-Pyridoxic acid, Isopyridoxal, and Pyridoxamine phosphate) content were higher in PBBT relative to PT (Table 1). Similar to our study, *A. solani* infection resulted in 37% reduction of VB6 content in *Arabidopsis*^92^. VB6 contents in *Arabidopsis* were lower in response to *Pst* DC3000 or *B. cinerea* infection^93^. In contrast, *B. cinerea* infection resulted in 53% increase in VB6 content in tomato plants^91^. Despite being contradictory, these results suggest an important role for VB6 in plant ROS management and pathogenicity; the up or down regulation of VB6 might be plant species specific.

This study provides the most comprehensive information to date on the metabolites involved in pea response to *A. euteiches* infection, with or without biocontrol bacterium inoculation. A wide range of metabolites, including polyunsaturated fatty acids, phenylpropanoids, and amino acids were upregulated in pathogen-treated pea, while being mostly at control levels in pea treated with the pathogen + bacterium. These compounds are likely to provide resistance to aphanomyces root rot in pea by acting as signaling molecules to initiate defense responses or as antimicrobial compounds. Many metabolites being at control levels in PBBT suggests that bacterial suppression of the pathogen resulted in the pea not needing to activate immune responses. The results of this study could facilitate the development of varieties and biocontrol agents (for example, by targeting the ethylene biosynthetic pathway, flavonoids, or arachidonic acid) that could improve food production and sustainability. Further studies to understand the precise role of defense-related metabolites in providing resistance against aphanomyces root rot, as well as to unravel detailed mechanisms of bacterium-mediated suppression of plant pathogens would benefit translational research.

### Supplementary Information

Supplementary Legends.

Supplementary Figure S1.

Supplementary Figure S2.

Supplementary Table S1.

Supplementary Table S2.

Supplementary Table S3.

Supplementary Table S4.

Supplementary Table S5.

Supplementary Table S6.

## Data Availability

All data generated or analysed during this study are included in this manuscript and its supplementary information files.
