# Author Correction: Deciphering *Aphanomyces euteiches*-pea-biocontrol bacterium interactions through untargeted metabolomics

**DOI:** 10.1038/s41598-024-66737-z

**Published:** 2024-07-10

**Authors:** Zakir Hossain, Shuang Zhao, Xian Luo, Kui Liu, Liang Li, Michelle Hubbard

**Affiliations:** 1 Swift Current Research and Development Centre, Agriculture and Agri-Food Canada, 1 Airport Road, Swift Current, Saskatchewan, S9H 3X2 Canada; 2 Department of Chemistry, University of Alberta, Edmonton, AB T6G 2G2 Canada

Correction to: *Scientific Reports* 10.1038/s41598-024-52949-w, published online 17 April 2024

Xian Luo was omitted from the author list in the original version of this Article.

The Author Contributions section now reads: “Z.H.: Conceptualization, Methodology, Investigation and analysis, Writing—original draft preparation, Writing—reviewing and editing. M.H.: Conceptualization, Methodology, Writing—reviewing and editing. K.L.: Methodology, Writing—reviewing and editing. L.L.: Methodology, Writing—reviewing and editing. S.Z.: Investigation and analysis. X.L.: Investigation and analysis.”

The original Article and the Supplementary Information have been corrected.

