# Supplementary material for: Deciphering Aphanomyces euteiches-pea-biocontrol bacterium interactions through untargeted metabolomics
Source: Sci Rep. 2024 Apr 17;14:8877. doi: 10.1038/s41598-024-52949-w (PMC11024177; doi:10.1038/s41598-024-52949-w)
Supplement: Supplementary file 1 — Supplementary Legends. [file 41598_2024_52949_MOESM1_ESM.docx]

**Supplementary materials**

**Supplementary Figure 1** Volcano plots showing the comparison of the levels of metabolites in roots between control and BBT (a) and control and PBBT (b). Metabolites with an FDR-corrected *p*-value (q-value) of less than 0.05 and with a fold change greater than 1.5 (or less than 0.67) were considered as significantly different.

**Supplementary Figure 2** Heatmap representing the relative abundances of select immunity-related metabolites. Each panel (bar) within a treatment represents one sample. Green and red denote low and high abundance, respectively. Name of individual metabolites are included at the end of the row.

**Supplementary Table 1** List of peak pairs detected from CIL LC-MS measurement of the samples (experimental data with imputated value).

**Supplementary Table 2** List of peak pairs that changed significantly between PT and control.

**Supplementary Table 3** Binary comparison of metabolites with relevant pathway/panel between PT and control, PBBT and PT, control and BBT, and control and PBBT (identified with high confidence in tier 1 and tier 2).

**Supplementary Table 4** List of peak pairs that changed significantly between PBBT and PT.

**Supplementary Table 5** Metabolic pathways relevant to pea-*A. euteiches* interactions (PT vs. control).

**Supplementary Table 6** Metabolic pathways relevant to pea-*A. euteiches*-bacterium and pea-*A. euteiches* interactions (PBBT vs. PT).
